# Practical and Surgical Anatomy

**Published:** 1839-07

**Authors:** 


ART. III.—*Practical and Surgical Anatomy.* By W. J. ERASMUS WILSON, Lecturer on Practical and Surgical Anatomy and Physiology. *Illustrated with Fifty Engravings on Wood, by Bagg.—London,* 1838. 8vo, pp. 492.

THIS is one of that very useful class of works intended to assist the student in the commencement of his application to practical anatomy, and one which the experience of seven years in the dissecting-room, as a teacher of anatomy, has convinced the author that the student requires. His design has been to interest the beginner, generally, in the pursuit of anatomy, and to assist him in acquiring an intimate acquaintance with its details; and, throughout the work, the subject of anatomy is shown in its most interesting relations to surgery.

The business of dissection is defined as being to divide and turn aside the integuments and fasciæ; to free the muscles from their cellular tissue, separating them, so as to display the vessels and nerves which lie between; and tracing these last to their ultimate ramifications. To attain dexterity and confidence in performing surgical operations, it is well observed that every dissection on the dead body should be made with care and precision, the parts divided or sought for being the same in both cases, although under different circumstances. These remarks should be kept in mind by the youngest student. It occasionally happens, in the course of practice, that observations casually made in the early part of every practitioner's study become unexpectedly useful; and this circumstance, which sometimes occurs in the practice of medicine, is much more likely to occur in relation to anatomical and surgical facts, which make the strongest impression when presented to the eye of the student for the first time. Without the careful superintendence of an experienced person, or some work aspiring to be more than a mere nomenclature of parts, much of the time spent in the dissecting-room must be misspent, and serve no other purpose but to give a false appearance of industry, without any of the results of application.

Upon the whole, we look upon this as a very valuable addition to the guide-books of the anatomical student. The greatest pains appear to have been paid to its composition; the surgical notices have had the benefit of Mr. Liston's revisal; the subject of the anatomy of the liver is treated of with the aid of Mr. Kiernan's researches; the description of the anatomy of the testis and thymus gland, and of herniæ, was facilitated by the permitted inspection of Sir Astley Cooper's beautiful preparations; that of the deep perineal fascia, and of the compressor urethræ muscle, by similar aid from Mr. Guthrie's collection; the anatomy of the eye is made with the assistance of Mr. Dalrymple's demonstrations; and in comparative anatomy the author acknowledges the advantage of Dr. Grant's friendly instruction. The work is throughout illustrated with numerous woodcuts, which clear up all the difficulties and obscurities; and yet there seems to have been no disposition in the author to make his publication a picture-book, for not one unnecessary figure is introduced. We strongly recommend Mr. Wilson's book to our younger readers, whether students at hospitals or in the field of public or private practice.

